# Risk Perception of Natural and Human-Made Disasters—Cross Sectional Study in Eight Countries in Europe and Beyond

**DOI:** 10.3389/fpubh.2022.825985

**Published:** 2022-02-14

**Authors:** Moran Bodas, Kobi Peleg, Nathan Stolero, Bruria Adini

**Affiliations:** ^1^Department of Emergency and Disaster Management, School of Public Health, Faculty of Medicine, Tel-Aviv University, Tel-Aviv-Yafo, Israel; ^2^National Center for Trauma and Emergency Medicine Research, The Gertner Institute for Epidemiology and Health Policy Research, Sheba Medical Center, Tel Aviv, Israel

**Keywords:** risk perception, socio-cultural, resilience, disasters, PRISM

## Abstract

Each year, emergency and disaster situations claim a heavy toll in human lives and economic loss. Civilian populations that are more aware and prepared for emergencies are more resilient. The aim of this study was to explore similarities and differences in risk perception of emergencies and disasters across different societies and its association with individual resilience. A cross sectional study that explored attitudinal factors, as expressed by diverse samples of target countries across Europe and beyond, took place during the months of January-February 2021. Diverse samples (*N* ≥ 500) of adults from 8 countries (Italy, Romania, Spain, France, Sweden, Norway, Israel, and Japan) were engaged in this study. This study used the Pictorial Representation of Illness and Self-Measure (iPRISM) tool to assess risk perception. The results suggest that for the overall sample (*N* = 4,013), pandemics were the risk of which participants showed the highest concern, followed by critical infrastructure fail, social disturbance, natural hazards, and extreme weather events. It was found that religiosity is associated with risk perception, with highly religious and non-religious reporting elevated risk perception (*F* = 5.735, *df* = 2, *p* = 0.003), however country-specific analysis revealed that this finding varies depending on local contexts. The analysis also revealed differences in risk perception depending on age and type of risk. The results of this study present that there are commonalities and differences between societies across Europe and beyond concerning societal resilience at large, including risk perception. The dependency of risk perception on local context suggests that a regional-based approach for disaster risk reduction may be called for to adapt and adjust to local socio-cultural characteristics of each population.

## Introduction

Each year, emergency, and disaster situations claim a heavy toll in human lives and economic loss. According to the Research Center for the Epidemiology of Disasters (CRED), over the past twenty years, 7,348 disaster events were recorded, claiming the lives of ~1.23 million people and affecting a total of more than 4 billion people. In addition, these disasters led to a loss of ~US$ 2.97 trillion worldwide (1).

There is a general consensus among scholars that civilian populations that are more prepared for emergencies are more capable of better reacting during the materialization of varied adversities, making them more resilient (2, 3). Resilience is defined as the ability to adapt to stressful or traumatic situations, maintaining homeostatic psychological functioning despite the apparent risk factors for distress and impaired functioning (4).

According Weber et al. (5) resilience is positively predictive of disaster preparedness. Therefore, a vital component in individual resilience is household adjustment to emergencies (6). It is widely accepted that households engaging in preparedness activities are more resilient, due to both increased awareness and actual adjustments that contribute to the survivability of family members in the aftermath of disaster (7–12). Yet, the understanding of the factors associated with preparedness and resilience remain elusive, despite extended efforts to devise elaborated models to explain preparedness behavior (13–15).

One of the important factors effecting preparedness behavior, and subsequently individual resilience, is risk perception [e.g., (6, 15–17)]. In the context of disaster preparedness, risk perception entails the awareness, beliefs, and attitudes concerning the likelihood, severity, threat intrusiveness, and additional attitudinal factors that may reflect on the manner in which one perceives the risk posed by any given threat (6). Given the complexity of assessing the multitude of attributes associated with risk perception, some scholars claim that a more holistic, qualitative measure of risk perception could be beneficial in assessing this factor (18).

One example of such a measure is the Pictorial Representation of Illness and Self-Measure (PRISM) tool. Developed by Büchi and Sensky (19), PRISM is a visual metaphor measuring personally salient appraisals and attitudes. The online version of the tool (iPRISM) provides participants with a visual metaphor for the relationship between oneself and selected objects possibly associated with it (20).

Originally, the PRISM tool was developed to assess the perceived burden of illness-related suffering, however several studies were able to demonstrate its capacities to successfully measure various appraisals within clinical practice (20–23) and other fields, including threat perception of natural hazards (24) and other emergencies and disaster situations (18). In fact, a systematic review of 52 studies that utilized the PRISM tool to assess attitudes and appraisals of subject-object(s) relationships concluded that it is likely to have wide applications in assessing beliefs, attitudes, and decision-making (25).

The aim of this study was to explore similarities and differences in risk perception of emergencies and disasters across different societies, in Europe and beyond, and its association with individual resilience.

## Materials and Methods

### Study Type, Population, and Sampling

This is a cross sectional study that explored attitudinal factors as expressed by diverse samples of eight target countries including Italy, Romania, Spain, France, Sweden, Norway, Israel, and Japan. The study took place during the months of January-February 2021, in the midst of the COVID-19 pandemic.

In each country, the target population was the adult population of the country (>18 years). In order to maintain working frameworks and consistency across studies countries, a national diverse sample of at least 500 participants was obtained from each country. The data collection was done by an internet panel company that used the stratified sampling method, based on data published by the Central Bureau of Statistics from each country concerning age, gender and geographic locations. The countries were chosen to reflect varied populations' characteristics, including from Western and Eastern European countries, as well as two countries beyond the European Union (Japan and Israel).

### Tools and Variables

The primary tool used in this reported study for the assessment of individual perception of risk was a visual tool called the Pictorial Representation of Illness and Self-Measure (PRISM), which was developed by Büchi and Sensky (19). The digital version of the PRISM tool (iPRISM) presented to the participants a digital white rectangular board with a fixed yellow disk at the bottom right corner. The participants were informed that this yellow disk represents themselves, while the white board represents their current life. Participants were then asked to place colored disks representing different threats/risks. The main quantitative measure derived from iPRISM is the distance, in centimeters, between the centers of the “object” and “self” disks. Distance measurements ranged between 0 and 26 cm, with smaller values representing increasing association between the participant and the objects at hand. The primary advantage of the iPRISM tool is that is appraises risk perception visually in a universal language that transcends cultural differences. While PRISM was developed originally to assess the perceived burden of suffering due to illness, it has been demonstrated to successfully measure various appraisals both within clinical practices and beyond, and has been demonstrated to have a wide range of applications (25).

The choice to use the iPRISM tool in this study was done in light of the need to rapidly evaluate many components of risk perception (e.g., perception of likelihood, severity, and threat intrusiveness) in a short and easy-to-perform task. Participants were asked to place the color disks based on the perception of how likely the threat may materialize, how severely the consequences may be to myself or those close to me as well as their potential intrusiveness on my life. Consequently, the iPRISM tool in this study assessed the risk perception individuals reported for the following risks adopted from UNESCO's categorization: (1) Extreme weather (cyclones, heat-waves, flooding…), (2) Nature related events (earthquake, volcanic eruption…), (3) Social disruption (attacks), (4) Critical services dependencies (water, energy…), and (5) Pandemic (communicable disease). For each participant, a general risk perception index was generated by averaging the distance score concerning all five hazards. This index scored a Cronbach's alpha value of 0.759.

Another important outcome assessed in this study was individual resilience. This construct was assessed with a three items questionnaire on a Likert scale ranging between 1 (“Not true at all”) to 5 (“True nearly all the time”). The tool was based on the Connor-Davidson Resilience Short Scale, 2003—abbreviated (2-item) version. The tool that measures perceived individual resilience was used with the consent of the authors. An example of an item in this scale is "I am able to adapt when changes occur.” Considering the aims of the study, one item was added; “I know the basic emergency rules that I should follow in case of an emergency.” The index has a Cronbach Alpha score of 0.821 and was generated by computing the mean responses to all three items.

### Study Data and Data Collection

Data acquisition was conducted through the service of *iPanel*, a public opinions polling service in Israel. Since 2006, the iPanel provides an online platform for a wide variety of information collection services, including polls and public opinion surveys. It adheres to the stringent standards of the world association for market, social, and opinion researchers (ESOMAR). iPanel was contracted to computerize the online questionnaire in all eight languages and to sub contract local vendors in each country to facilitate the dissemination of the questionnaire and data collection in each participating country.

All data collected was obtained through responses provided by participants in each of the participating countries to an online anonymous questionnaire. Questionnaires were presented in eight languages: Spanish, Romanian, Swedish, Norwegian, Italian, Japanese, French, and Hebrew. Each language was used in its respective country. Data was collected into spreadsheets and was collated into a single database on which statistical analysis was conducted.

### Statistical Analysis

Statistical analysis was conducted using SPSS (ver. 27). The analysis included both descriptive and analytical methods. Prior to analysis, indices were generated and their reliability was assessed using Cronbach's Alpha. Chi-square test was used to evaluate difference in proportions of variables between groups. Independent samples *t*-Test or Mann-Whitney's *U* test were used to compare means between independent samples. Spearman *R* test was used to assess correlation between continuous variables. In all statistical analyses performed, a *p*-value of 0.05 or less was determined as statistically significant.

## Results

### Overall Risk Perception

The overall sample of this study included 4,013 participants from eight countries: Israel, Sweden, Norway, Romania, Spain, France, Italy, and Japan. No statistical significances were observed between samples concerning the proportion of gender and the mean age. Table 1 provides the complete socio-demographic breakdown of the studied samples.

**Table 1:** Socio-demographic breakdown of the studied sample (*N* = 4,013).

**Variable**	**Israel**	**Sweden**	**Norway**	**Romania**	**Spain**	**France**	**Italy**	**Japan**
	***N =* 504**	***N =* 504**	***N =* 500**	***N =* 500**	***N =* 502**	***N =* 503**	***N =* 500**	***N =* 500**
**Gender**								
Female	258 (51.1%)	247 (49.0%)	236 (47.2%)	253 (50.6%)	245 (48.8%)	247 (49.1%)	243 (48.6%)	245 (49.0%)
Male	246 (48.7%)	257 (51.0%)	264 (52.8%)	247 (49.4%)	257 (51.2%)	256 (50.9%)	257 (51.4%)	255 (51.0%)
**Age**								
Average ± SD	39.93 ± 14.10	39.84 ± 13.65	40.11 ± 13.65	38.76 ± 12.99	39.03 ± 12.60	40.16 ± 13.05	40.17 ± 12.72	39.97 ± 12.73
Up to 24 (“Gen Z”)	89 (17.7%)	78 (15.5%)	85 (17.0%)	84 (16.8%)	69 (13.7%)	64 (12.7%)	60 (12.0%)	67 (13.4%)
25–40 (“Millennials”)	179 (35.5%)	195 (38.7%)	168 (33.6%)	199 (39.8%)	220 (43.8%)	208 (41.4%)	206 (41.2%)	196 (39.2%)
41–56 (“Gen X”)	157 (31.2%)	165 (32.7%)	187 (37.4%)	158 (31.6%)	152 (30.3%)	163 (32.4%)	167 (33.4%)	169 (33.8%)
57 and above (“Boomers”)	79 (15.7%)	66 (13.1%)	60 (12.0%)	59 (11.8%)	61 (12.2%)	68 (13.5%)	67 (13.4%)	68 (13.6%)
**Religion**								
Christian- Protestant	0 (0.00%)	137 (27.2%)	142 (28.4%)	15 (3.0%)	12 (2.4%)	21 (4.2%)	9 (1.8%)	8 (1.6%)
Christian- Catholic	0 (0.00%)	39 (7.7%)	47 (9.4%)	37 (7.4%)	270 (53.8%)	202 (40.2%)	341 (68.2%)	10 (2.0%)
Christian- Other	0 (0.00%)	53 (10.5%)	74 (14.8%)	382 (76.4%)	20 (4.0%)	12 (2.4%)	10 (2.0%)	4 (0.8%)
Muslim	1 (0.2%)	33 (6.5%)	26 (5.2%)	4 (0.8%)	6 (1.2%)	23 (4.6%)	2 (0.4%)	2 (0.4%)
Jewish	491 (97.4%)	5 (1.0%)	2 (0.4%)	0 (0.0%)	2 (0.4%)	1 (0.2%)	0 (0.0%)	4 (0.8%)
Other	0 (0.00%)	18 (3.6%)	19 (3.8%)	18 (3.6%)	12 (2.4%)	17 (3.4%)	12 (2.4%)	130 (26.0%)
Atheist/No religion	12 (2.4%)	219 (43.5%)	190 (38.0%)	44 (8.8%)	179 (35.7%)	226 (44.9%)	126 (25.2%)	342 (68.4%)
**Religiosity**								
Highly religious	80 (15.9%)	62 (12.3%)	25 (5.0%)	33 (6.6%)	26 (5.2%)	29 (5.8%)	42 (8.4%)	21 (4.2%)
Religious	104 (20.6%)	157 (31.2%)	168 (33.6%)	309 (61.8%)	168 (33.5%)	132 (26.2%)	251 (50.2%)	76 (15.2%)
Not religious	320 (63.5%)	284 (56.3%)	307 (61.4%)	158 (31.6%)	307 (61.2%)	341 (67.8%)	207 (41.4%)	400 (80.0%)
**Family status**								
Coupled with children	285 (56.5%)	158 (31.3%)	150 (30.0%)	244 (48.8%)	244 (48.6%)	236 (46.9%)	223 (44.6%)	157 (31.4%)
Coupled w/o children	81 (16.1%)	152 (30.2%)	127 (25.4%)	69 (13.8%)	109 (21.7%)	110 (21.9%)	98 (19.6%)	65 (13.0%)
Single with children	36 (7.1%)	28 (5.6%)	48 (9.6%)	32 (6.4%)	28 (5.6%)	44 (8.7%)	20 (4.0%)	25 (5.0%)
Single w/o children	102 (20.2%)	166 (32.9%)	175 (35.0%)	155 (31.0%)	121 (24.1%)	113 (22.5%)	159 (31.8%)	253 (50.6%)
**No. children <18 y/o**								
Average ± SD	1.16 ± 1.63	0.77 ± 1.77	0.57 ± 1.21	0.59 ± 1.10	0.75 ± 1.02	0.84 ± 1.12	0.62 ± 1.20	0.42 ± 1.21
**Education**								
< K-12	52 (10.3%)	40 (7.9%)	40 (8.0%)	28 (5.6%)	6 (1.2%)	43 (8.5%)	27 (5.4%)	15 (3.0%)
K-12 diploma	105 (20.8%)	164 (32.5%)	124 (24.8%)	118 (23.6%)	67 (13.3%)	132 (26.2%)	211 (42.2%)	139 (27.8%)
Vocational	104 (20.6%)	96 (19.0%)	81 (16.2%)	22 (4.4%)	126 (25.1%)	90 (17.9%)	40 (8.0%)	48 (9.6%)
Bachelor's degree	160 (31.7%)	130 (25.8%)	160 (32.0%)	237 (47.4%)	220 (43.8%)	126 (25.0%)	73 (14.6%)	256 (51.2%)
Master's or above	83 (16.5%)	74 (14.7%)	95 (19.0%)	95 (19.0%)	83 (16.5%)	112 (22.3%)	149 (29.8%)	42 (8.4%)
**Income**								
Much below average	100 (19.8%)	99 (19.6%)	83 (16.6%)	29 (5.8%)	48 (9.6%)	50 (9.9%)	15 (3.0%)	125 (25.0%)
Below average	107 (21.2%)	94 (18.7%)	101 (20.2%)	83 (16.6%)	89 (17.7%)	94 (18.7%)	55 (11.0%)	98 (19.6%)
Average	138 (27.4%)	176 (34.9%)	195 (39.0%)	253 (50.6%)	264 (52.6%)	239 (47.5%)	308 (61.6%)	192 (38.4%)
Above average	119 (23.6%)	105 (20.8%)	96 (19.2%)	118 (23.6%)	95 (18.9%)	99 (19.7%)	80 (16.0%)	59 (11.8%)
Much above average	39 (7.7%)	27 (5.4%)	24 (4.8%)	16 (3.2%)	6 (1.2%)	21 (4.2%)	42 (8.4%)	22 (4.4%)
**Experience with disasters**								
Yes	45 (8.9%)	67 (13.3%)	75 (15.0%)	38 (7.6%)	62 (12.4%)	54 (10.7%)	40 (8.0%)	64 (12.8%)
No	389 (77.2%)	387 (76.8%)	386 (77.2%)	415 (83.0%)	406 (80.9%)	408 (81.1%)	446 (89.2%)	372 (74.4%)
Not sure	70 (13.9%)	50 (9.9%)	39 (7.8%)	47 (9.4%)	34 (6.8%)	41 (8.2%)	14 (2.8%)	64 (12.8%)

*Maximum missing per country per variable is 4 (0.8%)*.

**Figure 1:**
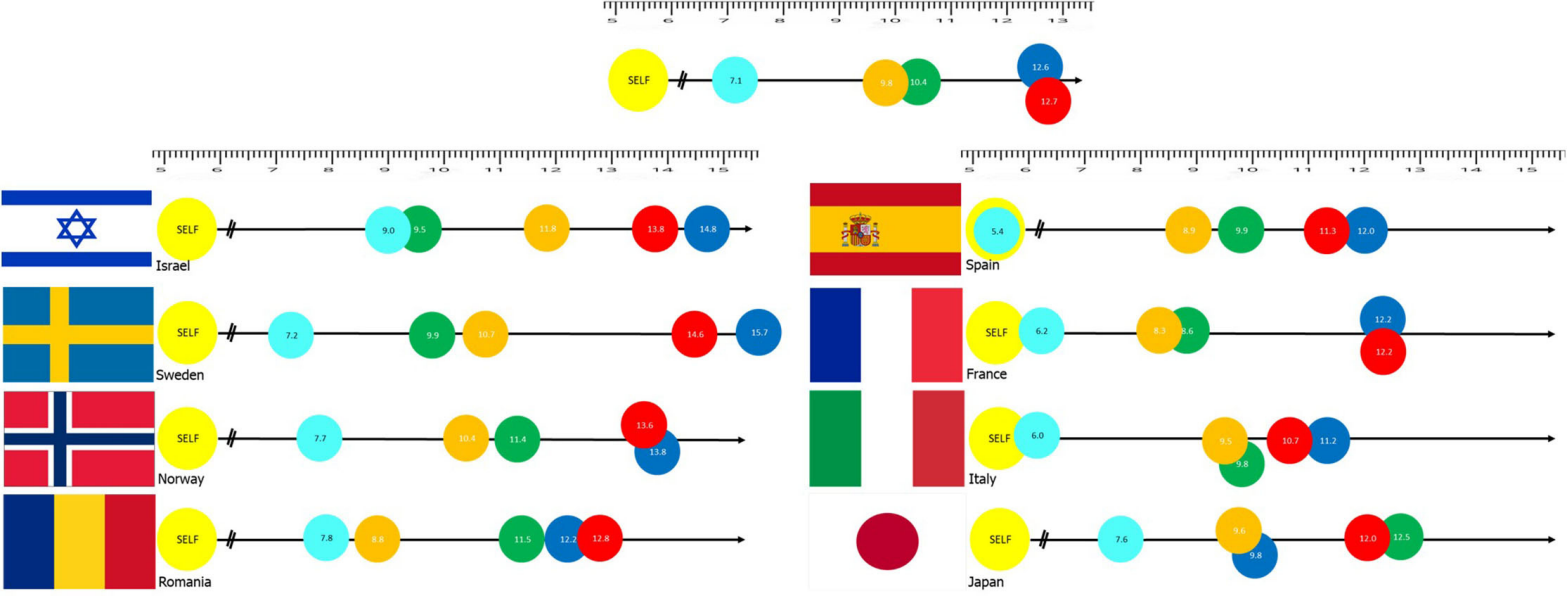
Results of the iPRISM tool assessing risk awareness through distances assigned by participants between themselves (yellow “SELF” disk) and specific risk objects [Light blue: Pandemics, Orange: Critical infrastructure fail (water, energy), Green: Social disruption (e.g., war), Blue: Natural Hazard (e.g., earthquakes), and Red: Extreme weather]. Top image is overall sample (*N* = 4,013). National samples are presented with their flag on the right of the image.

The assessment of individual perception of risk was conducted using the digital Pictorial Representation of Illness and Self-Measure (iPRISM) tool (see methods). The results suggest that for the overall sample, pandemics were the risk of which participants showed the highest concern with a mean distance of 7.11 (±5.40 SD) followed by critical infrastructure fail (9.76 ± 6.03 SD), social disturbance (10.39 ± 6.02 SD), natural hazards (12.57 ± 6.63 SD), and extreme weather events (12.72 ± 6.99) (see Figure 1).

### Country-Based Risk Perception

The results obtained from the iPRISM tool allow for a comparison between the different countries to assess differences in risk perception between cohorts of different nationalities. Figure 1 also provides an illustration to compare between risk perception analyses of the eight countries in this study. Patterns reported for risk perception in the general results were also re-examined in a country-by-country basis. Results suggest that countries demonstrate different patterns of correlations between risk perception and socio-demographic factors.

For example, in the overall sample (*N* = 4,013) the analysis suggests that highly religious (10.79 ± 5.34 SD) and non-religious (10.67 ± 4.31 SD) have the highest risk perception, compared with religious individuals (10.19 ± 4.44 SD), according to One-way ANOVA test (*F* = 5.735, *df* = 2, *p* = 0.003). *Post-hoc* Bonferroni's correction analysis suggest that this statistical significance is attributed to the difference between the non-religious and religious cohorts (mean difference = 0.48, *SE* = 0.15, *p* = 0.004) only. The country-specific analysis reveals the origin of this pattern. In Israel, there is a negative correlation between religiosity and risk perception. Highly religious individuals score a higher mean distance of the color disks (13.62 ± 4.95 SD), i.e., have a lower risk perception, compared to religious (12.59 ± 4.00 SD) and non-religious (11.00 ± 3.73 SD), according to One-way ANOVA test (*F* = 16.7, *df* = 2, *p* < 0.001). In Sweden, however, the picture is the opposite with non-religious individuals placing the disks farther (12.36 ± 4.20 SD) than religious (10.84 ± 4.15 SD) and highly religious people (10.48 ± 5.15 SD) (*F* = 8.80, *df* = 2, *p* < 0.001). In all other six countries, that data is statistically non-significant.

In the overall sample, age and risk perception index are not correlated (*p* = 0.09). However, in the country specific analysis, age and risk perception are correlated in Sweden [*R*_(504)_ = 0.107, *p* = 0.016] and Italy [*R*_(500)_ = 0.127, *p* = 0.004], which means that in these countries the older populations have a lower risk perception compared to younger ones. In the other countries, there is no such association between age and risk perception.

**Figure 2:**
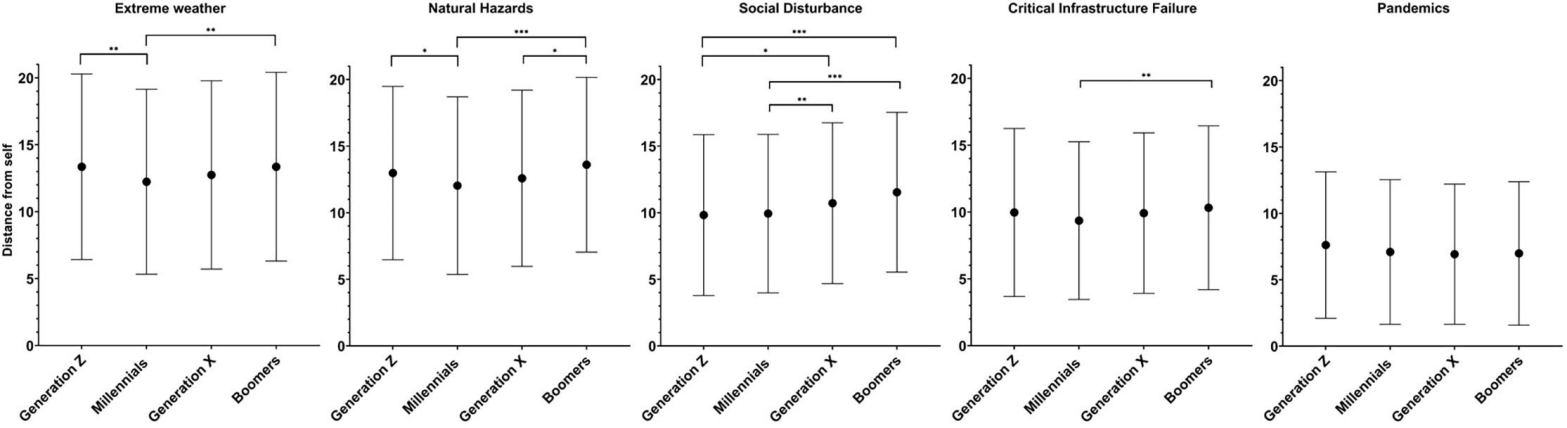
Results of the iPRISM tool assessing risk awareness through distances assigned by participants between themselves (yellow “SELF” disk) and specific risk objects across age groups (“Generation Z”: 18–24 years of age, “Millennials”: 25–40, “Generation X”: 41–56, and “Boomers” and earlier generations: 57 and above). ^*^p < 0.05, ^**^p < 0.01, ^***^p < 0.001.

Participants were categorized into four age groups (“Generation Z”: 18–24 years, “Millennials”: 25–40, “Generation X”: 41–56, and “Boomers” and earlier generations: 57 and above). In the overall sample, the comparison of risk perception across these categories present a significant difference between the groups, according to One-way ANOVA test (*F* = 8.195, *df* = 3, *p* < 0.001); Millennials score the highest risk perception (i.e., lowest distance scores) (10.14 ± 4.45 SD) compared with Generation X (10.58 ± 4.45 SD), Generation Z (10.75 ± 4.34 SD), and Boomers (11.16 ± 4.50 SD). *Post-hoc* Bonferroni's correction analysis suggest that this statistical significance is attributed to the difference between “Millennials” and all other categories, namely “Gen Z” (mean difference = −0.62, *SE* = 0.21, *p* = 0.024), “Gen X” (mean difference = −0.44, *SE* = 0.17, *p* = 0.044), and “Boomers” (mean difference = −1.02, *SE* = 0.22, *p* < 0.001). Analysis of each hazard separately reveals additional findings (see Figure 2). While all age groups perceived the pandemic threat as the highest risk (placed the disc similarly close to oneself), differences between nationalities were identified concerning the other types of risks. For instance, Millennials view the risk posed by natural hazards and extreme weather more seriously than Generation Z and boomers. Younger participants view social disturbance more seriously than older participants. See complete data in Figure 2.

Risk perception was correlated with individual resilience in all countries (Spearman R ranges from 0.216 in Israel to 0.416 In Norway, *p* < 0.001 in all). The correlation suggests that placing the disks farther away from oneself signifies a higher level of individual resilience. Respondents with a lower perception of individual resilience placed the disks closer to themselves, i.e., perceived the risk as being higher, compared to individuals with a higher perceived individual resilience. This phenomenon is shared across societies.

## Discussion

This study provides a unique glance into risk perception in eight different societies representing different cultures, using the iPRISM tool. This intuitive measurement of individual concern and awareness over different risks incorporates different modalities of risk perception and allows an overview of individual threat appraisal. The results of this study demonstrate that societies have different risk perceptions depending on local contexts.

Socio-cultural contexts lead to varied risk perceptions and consequently, to different ways of preparing for each type of hazard. For example, while Romania and Malta share similar risks, Romanian people tend to base their emergency preparedness on individual collection of information from the authorized bodies; while Maltese people tend to base their preparedness on social activities (26). Cultural contexts such as values, traditions, technological literacy, responsibilities attributed to varied sectors of the population and more, impact on both risk perceptions and emergency preparedness (26, 27).

Not surprisingly, risk perception, as displayed in the study, reflects the public's tendency to focus on risks that significantly disrupt their routine while other risks are considered much less concerning. As expected, the global COVID-19 pandemic was perceived by the respondents from all countries as the most concerning. Nonetheless, the level of this risk was perceived in different intensities among the respondents from the respective countries. People from the three Western European countries (Spain, France and Italy) perceived this risk as more threatening to them, compared to individuals from the Scandinavian countries (Sweden and Norway) as well as from Romania and Japan, and even more so from Israel. These diverse findings may be associated with the management of the pandemic in the respective countries early into the COVID-19 pandemic. The levels of infectivity of COVID-19 and their subsequent impact on morbidity and mortality were highest in those European countries (28, 29), while the levels of vaccine hesitancy distanced the capacity to achieve herd immunity (30). Furthermore, those three countries previously experienced surge capacities of COVID-19 patients that overwhelmed their healthcare systems, leading to potential decreased confidence and trust in their leadership (31, 32).

What was interesting to note was that the respondents from most of the countries perceived all other risks as being much less relevant or threatening to their lives (and thus, placed the other risks at a distance from the current threat), while only two countries (Israel and Sweden) perceived an additional risk to also be substantial (and thus, placed another risk relatively close to the pandemic risk). Israeli respondents perceived social disruption as a similarly relevant risk to that of the pandemic, reflecting the growing concern over political instability that the country is experiencing in recent years (33). Swedish respondents also perceived social disruptions as the second highest risk; this may be related to the current societal tension that exists due to the refugee crisis (34, 35). The respondents from all other societies ranked infrastructure failures as the second most relevant risk, but the Romanian respondents indicated a higher level of concern regarding this risk, most probably as this threat has been in major focus in Romania in recent decades (36, 37).

Contrary to the studied societies, Japanese respondents were more concerned with natural hazards. Being situated in the “Ring of Fire,” a geographical area of the Earth's crust known to be prone to earthquakes, most probably causes Japanese to be much more concerned about natural hazards, such as earthquakes, than Europeans (38).

Arguably, the findings here echo the notions raised that disaster risk reduction and planning should be context driven and adapt to local risk patterns. In contrast to the All-Hazards Approach, which is the current dogma in disaster planning, the Top-Hazards Approach (39) assumes that risks are inherently different and pose different challenges to risk planners. COVID-19 is a prime example to showcase this argument (40). The results of this study show that public opinions support the differential perception of risks according to local contexts, that include political, social and medical considerations.

This is well-displayed in the contradictory trends that were found in different countries concerning the association between level of religiosity and risk perception. While in Israel, highly religious individuals present lower levels of risk perception compared to secular or somewhat religious people, the opposite trend was found among the Swedish respondents. Ultra-orthodox (highly religious) populations in Israel, for example, were reluctant to adhere to the public health measures that were instructed by the authorities as they disrupt their “observances” and at times may even contradict guidelines relayed by the religious leadership (41). This in turn increases mistrust in the authorities, and consequently leads to a tendency to disregard the risk or decrease its perceived endangerment to them (42). This lower risk perception was evident even when the prevalence of COVID-19 was higher in the ultra-Orthodox communities compared to religious or secular populations. Conversely, in Sweden ‘*religion does not constitute the major part of people's existential orientation system and therefore is not integral to people's everyday lives*” (43). Furthermore, the prevalent policy in Sweden was based on recommendations to the public, that were not mandatory but rather sought their personal judgment and commitment (44). Accordingly, as there was no contradiction between the religious norms and traditions to the requested public health measures, religious individuals were able to harmonize between their religious beliefs customs and the expected health behavior (45).

The findings of this study also provide an interesting look into differences in risk perception and perception across age groups and generations. The data suggests mixed results with regards to the effect age has on risk perception. For example, younger individuals seem to be more concerned about social disturbance than older individuals are. However, when it comes to extreme weather and natural hazards, millennials, who are young parents these days, seem to be more concerned. This finding echoes other findings reported in the literature, for example by Gray et al. (46). Although this study found no differences across age groups regarding the pandemic threat, other studies found that millennials demonstrated higher levels of concerns compared to older individuals (47, 48).

The higher levels of risk perception among millennials compared to other age groups may be explained by their being more vulnerable to economic (risk of losing their employment) or social disorders (vital services such as the education system for their young children may be compromised) (49). In contrast, boomers have lesser concerns for their financial stability (many may already be pensioned) and their financial literacy appears to be higher compared to Gen Z and other age groups (50). This is accentuated by the boomers' tendency to perceive their being under risk to a lesser level compared to other age groups, even concerning the pandemic, despite the fact that they are considered more vulnerable to the virus. This finding is in line with the results reported by Brafman et al. (51) that found that during the COVID-19 pandemic younger populations (Generation Z and Millennials) presented higher levels of loneliness and mental health problems than those of the older generations.

Lastly, the results of this study indicate that participants reporting increased individual resilience are less concerned about selected risks. This finding provides additional support to those reported in the literature about the association between risk perception and preparedness behavior, and subsequently resilience [e.g., (6, 15–17)]. Antronico et al. (52) found that enhancing communication and information, as well as involving individuals in actions targeted to raise preparedness to respond to potential hazards increase resilience as well as risk perceptions and governance. Furthermore, Qing et al. (53) have presented that risk perceptions serve as a mediator between preparations that individuals implement to manage varied hazards and their levels of resilience.

Nevertheless, an alternative explanation may be provided to this finding, namely the optimism bias. For example, Paton (13) describes the optimism bias as leading individuals to assess unrealistically their level of preparedness and resilience as high, despite it being relatively low in actuality. Other explanations may include the victimization model (54), which argues that under frequent and ongoing exposure to threat, people develop skewed perception over resilience and preparedness. Additional research may be warranted to explore in more details the fundamentals of the association between risk perception and reported individual resilience.

## Limitations

This study has some limitations. First, technical constraints limited the national samples sizes to 500 in each country. While in some countries this sample size is adequate to provide a representative sample of the entire population (e.g., Israel, in regard to the Jewish population), in other participating countries it may be difficult to fully cover all different groups in the society. Therefore, generalization of the conclusions of this study should be done with caution and more in-depth analysis in each participating country is called-for to substantiate the local findings. Second, this study was performed online. Accessing participants through online channels proves to be a very rapid way of collecting response in a wide geographical distribution. Nonetheless, it limits the conclusions to participants with the minimal set of skills needed to perform the questionnaire online. Therefore, findings should be limited to individuals with adequate digital literacy and access to digital tools. Lastly, as is the case with other cross sectional studies, this study assessed attitudes and opinions in a certain point in time, and more so, during a prolonged global pandemic. Fluctuations in circumstances surrounding the study could register a temporal effect on individuals' perceptions. Therefore, the conclusions of this study are relevant to the point in time in which they were collected.

## Conclusions

The results of this study show that there are commonalities and differences between societies across Europe and beyond concerning risk perception of disasters. This study provides a unique glance into risk perception using the iPRISM tool. This intuitive measurement of individual concerns and awareness over different risks incorporates different modalities of risk perception and allows an overview of individual threat appraisal. The conclusion of this study in this regards is that societies have different risk perceptions depending on local contexts. Therefore, instead of adopting a global approach to resilience promotion, a regional-based approach is needed to adapt and adjust to local socio-cultural contexts.

## Data Availability Statement

The raw data supporting the conclusions of this article will be made available by the authors, without undue reservation.

## Ethics Statement

The studies involving human participants were reviewed and approved by the Ethical Committee of the Tel-Aviv University (Approval No. 0002377-1 dated November 25, 2020). In addition, this study was approved by the Ethical Committee of the Norwegian Research Council. Subsequently to this approval, the study was granted exemption from further approvals in each participating country. The patients/participants provided their written informed consent to participate in this study.

## Author Contributions

MB, KP, NS, and BA contributed to conception and design of the study. MB organized the database, performed the statistical analysis, and wrote the first draft of the manuscript. BA wrote sections of the manuscript. All authors contributed to manuscript revision, read, and approved the submitted version.

## Funding

The research leading to these results has received funding from Horizon 2020, the European Union's Framework Programme for Research and Innovation (H2020/2014-2020) under grant agreement n° 882850.

## Conflict of Interest

The authors declare that the research was conducted in the absence of any commercial or financial relationships that could be construed as a potential conflict of interest.

## Publisher's Note

All claims expressed in this article are solely those of the authors and do not necessarily represent those of their affiliated organizations, or those of the publisher, the editors and the reviewers. Any product that may be evaluated in this article, or claim that may be made by its manufacturer, is not guaranteed or endorsed by the publisher.
