# Supplementary material for: Risk Perception of Natural and Human-Made Disasters—Cross Sectional Study in Eight Countries in Europe and Beyond
Source: Front Public Health. 2022 Feb 14;10:825985. doi: 10.3389/fpubh.2022.825985 (PMC8896349; doi:10.3389/fpubh.2022.825985)
Supplement: Supplementary file 1 [file Data_Sheet_1.PDF]

Engage Society for
Risk Awareness and Resilience

https://www.project-engage.eu/

Survey T1.2

In the following questions when we say "community", we mean the people living in your area of residence.

Social norms and sense of communality

	**In my area of residence:**	Strongly Disagree	Disagree	Neither agree or disagree	Agree	Strongly Agree
1.	There is mutual assistance and people care for one another.	1	2	3	4	5
2.	I am proud to tell others where I live.	1	2	3	4	5
3.	I can count on people in my community to help me in a crisis.	1	2	3	4	5
4.	I have a sense of belonging to my community.	1	2	3	4	5
5.	Residents in my community trust each other.	1	2	3	4	5
6.	The members of my community participate along with the first responders and authorities to facilitate the recovery.	1	2	3	4	5

Coping skills, styles, and resources

	**Consider how well the following statements describe your behavior and actions:**	Does not describe me at all	Does not describe me	Neutral	Describes me	Describes me very well
7.	I look for creative ways to alter difficult situations.	1	2	3	4	5
8.	Regardless of what happens to me, I believe I can control my reaction to it.	1	2	3	4	5
9.	I believe I can grow in positive ways by dealing with difficult situations	1	2	3	4	5
10.	I actively look for ways to replace the losses I encounter in life	1	2	3	4	5

Actual preparedness

	**Do you have any of the following items in place in/for your home?**	Yes	No	Not sure	Not relevant
11.	A working transistor radio with spare batteries or smartphone with portable charger	1	0	998	999
12.	At least 3 liters of water per person in your family	1	0	998	999
13.	A 4-day supply of non-perishable food items for each person in your family	1	0	998	999
14.	A fire extinguisher	1	0	998	999
15.	Medical needs for family members	1	0	998	999
16.	Photocopy/backup of important documents	1	0	998	999
17.	List of vital phone numbers of family members	1	0	998	999
18.	A household emergency plan	1	0	998	999

	To the best of your knowledge, do you have any of the following items in place at your community?	Yes	No	Not sure	Not relevant
19.	Common shelters for people to be protected if need be	1	0	998	999
20.	A community-based assistance network in which members of the community help each other during crises	1	0	998	999
21.	Good access to emergency services during crises	1	0	998	999
22.	A community emergency plan	1	0	998	999

Perception of trust & responsibility

	**Please rate the level of trust you have in the following institutions:**	Not at all	A little	Somewhat	Much	Very much
23.	The government	1	2	3	4	5
24.	The civil Defense/protection	1	2	3	4	5
25.	The local authority	1	2	3	4	5
26.	The first responders	1	2	3	4	5
27.	The politicians	1	2	3	4	5
28.	The media	1	2	3	4	5
29.	Non-Governmental Organizations (NGOs)	1	2	3	4	5
30.	Health services	1	2	3	4	5
	**To what extent do you think the following are responsible to prepare to emergencies?**	Not at all	A little	Somewhat	Much	Very much
31.	The government	1	2	3	4	5
32.	The civil Defense	1	2	3	4	5
33.	The local authority	1	2	3	4	5
34.	The health services	1	2	3	4	5
35.	Your community	1	2	3	4	5
36.	Yourself and your family	1	2	3	4	5

Individual Resilience

	To what extent do you agree with each of the following statements:	Not true at all	Rarely true	Sometimes true	Often true	True nearly all the time
37.	[redacted]*	0	1	2	3	4
38.	[redacted]*	0	1	2	3	4
39.	I know the basic emergency rules that I should follow in case of an emergency	0	1	2	3	4

* *Per agreement with the author of the original tool (The Connor-Davidson Resilience Scale ©), these items were omitted from this public version of the questionnaire*

Communication

	Consider how well the following statements describe your behavior and actions	Not at all	A little	Somewhat	Much	Very much
40.	I believe I have the ability to provide first responders with important information during an emergency	1	2	3	4	5

	What is the likelihood that you will use the following media sources for information during a disaster?	Not at all	A little	Somewhat	Much	Very much
41.	Mobile phones (e.g., text, calls, instant messaging)	1	2	3	4	5
42.	Social media (e.g., Facebook, Twitter, Instagram)	1	2	3	4	5
43.	Television					
44.	Other electronic communications (email, discussion boards, websites)	1	2	3	4	5
45.	Printed communications (newsletter, leaflets, letters)	1	2	3	4	5
46.	Face-to-face or personal communication	1	2	3	4	5

National resilience

	To what extent do you agree with each of the following statements relating to your country in the context of emergency preparedness:	Strongly Disagree	Disagree	Neither agree nor disagree	Agree	Strongly Agree
47.	I believe that my government will make the right decision during a time of crisis.	1	2	3	4	5
48.	I have full confidence in the ability of the emergency services of my country to protect our population.	1	2	3	4	5
49.	My society has coped well with past crises.	1	2	3	4	5
50.	I am optimistic about the future of my country.	1	2	3	4	5
51.	In my society, there is a high level of social solidarity (mutual assistance and concern for one another).	1	2	3	4	5
52.	In my society, there is a reasonable level of social justice.	1	2	3	4	5

53.	I have full faith in the ability of my country's health system to care for the population in crisis.	1	2	3	4	5
54.	I have complete confidence in the ability of my government to take care of all aspects relevant to overcoming crises.	1	2	3	4	5

Communication needs

	**In case of a disaster (e.g., flood, earthquake, pandemic), how important are each of the following information needs to you?**	Not important at all	Not Important	Somewhat important	Important	Very important
55.	To receive information that can help me talk about the situation with others.	1	2	3	4	5
56.	To receive information that can help me feel as part of the community/nation.	1	2	3	4	5
57.	To receive information that can distract my thoughts from the situation.	1	2	3	4	5
58.	To receive credible information.	1	2	3	4	5
59.	To be able to actively share information with the authorities/relevant organizations acting on the situation.	1	2	3	4	5
60.	To receive information that can make me feel positive emotions (e.g., happiness, amusement, joy).	1	2	3	4	5
61.	To receive information as fast as possible.	1	2	3	4	5

Digital Literacy

	**To what extent do you agree or disagree with the following statements:**	Strongly Disagree	Disagree	Neither Agree nor Disagree	Agree	Strongly Agree
62.	Learning to use new mobile apps or websites is easy for me	1	2	3	4	5
63.	Using mobile apps or websites to find information is easy for me	1	2	3	4	5

Demographics

A few questions for statistical analysis purposes only. All information is completely anonymous.

64. Gender: Male / Female / Other
65. Year of birth: ________
66. Country _________
67. Place of residence: _____________
68. Familial status:
    - Coupled with children
    - Coupled without children
    - Single with children
    - Single without children
69. Number of children under 18: _____

70. Affiliation to religion:
    - Christian - Protestant
    - Christian - Catholic
    - Christian - Other
    - Moslem
    - Jewish
    - Other religion
    - Atheist
71. Level of religiosity
    - Highly religious
    - Religious
    - Not religious
72. Education
    - Less than full high school education (K-12)
    - Full high school education (K-12)
    - Vocational education
    - Bachelor's degree
    - Master's degree or higher
73. Income
    - Much below average
    - A little below average
    - Average
    - A little above average
    - Much above average
74. Have you been personally exposed in the past five years to a significant disaster risk?
    - Yes
    - No
    - Don't know / not sure
75. To which community do you feel belong (e.g., local neighborhood, ethnic minority, cultural community, or other), please specify: ___________________
